# Chronic Invasive Aspergillosis caused by *Aspergillus viridinutans*

**DOI:** 10.3201/eid1508.090251

**Published:** 2009-08

**Authors:** Donald C. Vinh, Yvonne R. Shea, Pamela A. Jones, Alexandra F. Freeman, Adrian Zelazny, Steven M. Holland

**Affiliations:** National Institutes of Health, Bethesda, Maryland, USA (D.C. Vinh, Y.R. Shea, P.A. Jones, A. Zelazny, S.M. Holland); National Cancer Institute-Frederick, Frederick, Maryland, USA (A.F. Freeman); 1 These authors contributed equally to this article.

**Keywords:** aspergillosis, chronic, contiguous, disseminated, invasive, Aspergillus, viridinutans, fumigatus, fungi, dispatch

## Abstract

*Aspergillus viridinutans*, a mold phenotypically resembling *A. fumigatus*, was identified by gene sequence analyses from 2 patients. Disease was distinct from typical aspergillosis, being chronic and spreading in a contiguous manner across anatomical planes. We emphasize the recognition of *fumigati*-mimetic molds as agents of chronic or refractory aspergillosis.

*Aspergillus fumigatus* is the most common cause of invasive aspergillosis afflicting various immunocompromised patients. We report 2 cases of documented invasive disease in children in the United States caused by *A. viridinutans*, a *fumigati*-mimetic mold, which produced clinical manifestations distinct from *A. fumigatus*.

## The Patients

**Figure 1:**
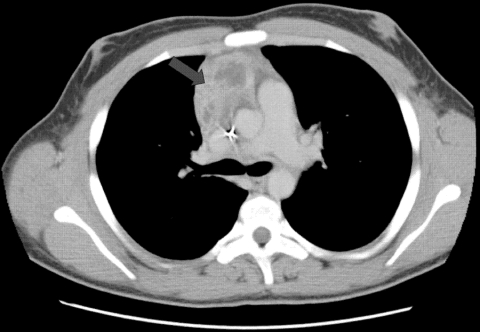
Computed tomographic scan of thorax showing extension of infection with *Aspergillus* *viridinutans* into mediastinal structures (arrow).

Patient 1 was a 14-year-old boy with p47*^phox^*-deficient chronic granulomatous disease. He had *Staphylococcus aureus* liver abscesses at ages 5 and 10 years, *Burkholderia cepacia* complex pneumonia at age 6 years, and *Serratia marcescens* pneumonia at age 12 years. In February 2004, while the patient was receiving itraconazole prophylaxis, right-sided chest pain and fevers developed. A computed tomography (CT) scan showed a 2-cm right middle lobe nodule adjacent to the cardiac border and mediastinal lymphadenopathy abutting the superior vena cava and anterior pericardium (Figure 1). Lymph node biopsy yielded a mold morphologically identified as *A. fumigatus*. Treatment with voriconazole was initiated. Repeat imaging 1 week later showed slight enlargement of the mediastinal mass. One month later, there was continued enlargement of the lung nodule and mediastinal adenopathy with necrosis. Caspofungin was added. Two weeks later, a new right middle lobe infiltrate was noted. Antifungal therapy was changed to posaconazole. Over the next 2 months, there was expansion of the right lung infiltrates and lymphadenopathy. Treatment was modified to posaconazole and caspofungin. Serial imaging over the next 3 weeks showed regression of the lung consolidations and mediastinal mass. Four months later, with ongoing resolution of the thoracic disease, the patient began receiving a maintenance dosage of posaconazole. As of 5 years later, he had experienced no recurrence.

**Figure 2:**
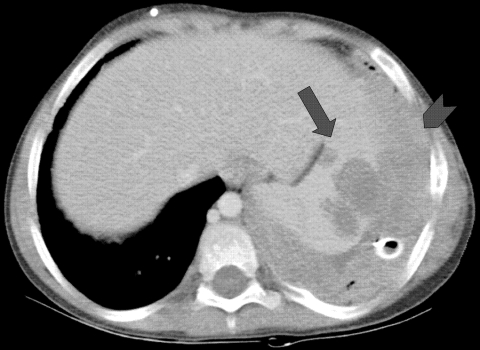
Computed tomographic scan showing infection with *Aspergillus viridinutans*, originating in the lungs, extending into the diaphragm (arrowhead), and producing hypodense splenic lesions (arrow).

Patient 2 was an 8-year-old boy with hyperimmunoglobulin-E (Job) syndrome due to mutation in signal transducer and activator of transcription-3 in whom a right-sided pulmonary abscess developed and failed to improve after 1 month of antibacterial therapy. New left lung nodules were biopsied and specimens yielded a mold morphologically identified as *A. fumigatus*. During 3 months of treatment with voriconazole, the bilateral pulmonary lesions cavitated. Two months later, left lower lobe wedge resection yielded the same mold. Diaphragmatic injury required primary closure with sutures. One month later, enlargement of the residual left lung lesions prompted change in therapy to amphotericin B and caspofungin. Following the patient's transfer to the National Institutes of Health, imaging showed left apical and lower lobe cavitary masses, left pleural mass and effusion, and a right upper lung cavity with nodule. Samples taken from the left lower cavity and pleura demonstrated septated branching hyphae; specimens grew a mold resembling *A. fumigatus*. Amphotericin B–related nephrotoxicity prompted change of treatment drug to posaconazole. Tissues obtained through subsequent surgical debridements of lung, pleural, diaphragmatic, and subpulmonic abscesses, left lower lobe segmentectomy, and decortication all grew a mold morphologically consistent with *A. fumigatus*. The patient’s medical regimen was changed to posaconazole, caspofungin, and flucytosine. Persistent pleural tube drainage yielded the same fungus, despite treatment with amphotericin B and granulocyte intrapleural infusions. Systemic granulocyte infusions, adjunctive granulocyte-colony stimulating factor, and interferon-γ led to transient stabilization of disease. However, progressive splenic abscess formation contiguous with the diaphragm was noted (Figure 2). Seven months later, Guillain-Barré syndrome without a clear cause developed in the patient. The patient died of progressive respiratory failure with aspiration of abscess cavity content. Autopsy demonstrated extensive fungal abscesses in the lungs and left pleural space that extended into the airways, diaphragm, and spleen.

Isolates were initially identified by morphologic criteria using standard clinical mycology laboratory media and incubation conditions. Subsequently, multilocus sequence analyses were performed and targeted the internal transcribed spacer (ITS) 1 and 2 regions flanking 5.8S rDNA, (partial) β-tubulin gene (*benA*), and (partial) calmodulin gene as previously described (*1*–*4*). Sequences were assembled by using Lasergene version 7.0 (DNASTAR; www.dnastar.com), compared with sequences in GenBank by using BLAST (www.ncbi.nlm.nih.gov/BLAST), and realigned relative to best-matched sequences by using MegAlign (DNASTAR). Sequencing of ITS assigned the isolates to *Aspergillus* section *Fumigati* but could not provide further intrataxon discrimination. The β-tubulin sequence from patient 1 had 96.3% and 99.5% similarity with *A. viridinutans* type strain IMI062875T (AF134779) and strain IMI182127 (AF134777), respectively. Three isolates from patient 2 were tested, demonstrating 96.3% and 98.4% similarity with *A. viridinutans* type strain CBS127.56T (AF134779) and NRRL6106 (AF134778), respectively. The calmodulin sequences from patient 1 and patient 2 demonstrated 99.8% and 99.0% similarity, respectively, to *A. viridinutans* IMI182127 (DQ094888). The 2 patients’ isolates were 98.6% similar to each other. The strains were designated NIHAV1 (from patient 1) and NIHAV2 (from patient 2). The deduced amino acid sequence from NIHAV1 had 100% similarity with strain IMI182127 (AF134777); NIHAV2 differed by 1 aa with strain NRRL6106 (AF134778). NIHAV1 and NIHAV2 differed by 1 aa (98.8% similarity). Sequences obtained for NIHAV1 *benA*, NIHAV1 calmodulin, NIHAV2 *benA*, and NIHAV2 calmodulin have been deposited in GenBank under accession nos. GQ144441, GQ144442, GQ144440, and GQ144443, respectively.

Antifungal drug susceptibility testing was performed by broth microdilution at the Fungus Testing Laboratory, University of Texas Health Sciences Center, using Clinical Laboratory Standards Institute’s contemporary method M38A (Table). Isolates underwent extended incubation to ensure sufficient conidia for testing. Although no clinical interpretive breakpoints are established, most clinical isolates identified as *A. fumigatus* have MICs to amphotericin B and voriconazole <0.5–1 mg/L (*5*–*7*). The *A. viridinutans* isolates, however, demonstrated significantly higher MICs to amphotericin B and voriconazole, agents recommended as first-line therapy for treatment of aspergillosis (*8*). This in vitro resistance may explain in part the refractory disease seen clinically. Furthermore, synergistic studies of isolates from patient 2 with the combination of either posaconazole and terbinafine or amphotericin B and terbinafine produced an indifferent effect.

**Table:** Antifungal drug susceptibility results of *Aspergillus viridinutans* isolates relative to *A. fumigatus* reported at 48 hours*

Isolate	Amphotericin B MIC, mg/L	Itraconazole MIC, mg/L	Voriconazole MIC, mg/L	Posaconazole MIC, mg/L	Caspofungin MEC, mg/L	Terbinafine MIC, mg/L
Patient 1	4	1	1	0.06	0.25	ND
Patient 2†	2–8	8	2–4	<0.016–0.5	0.06–0.25	0.125–1
*A. fumigatus* B-5233‡	0.5	0.5	0.5	0.125	0.25	2

*MEC, minimal effective concentration; ND, not determined.  †Composite results of 3 isolates from the patient.  ‡Clinical isolate from patient with a fatal case of aspergillosis (courtesy of K.J. Kwon-Chung, National Institute of Allergy and Infectious Diseases, Bethesda, MD, USA).

## Conclusions

Differentiation of species within *Aspergillus* section *Fumigati* using phenotypic features alone is difficult. Our isolates resembled *A. fumigatus* but demonstrated slower growth at 37°C and markedly reduced sporulation. Based on multilocus sequence analyses, however, these isolates are *A. viridinutans*, a mold phenotypically resembling but phylogenetically distinct from *A. fumigatus*. Although the ITS region failed to distinguish these isolates from *A. fumigatus*, weak intrataxon discriminatory capacity has been previously reported for this locus (*9*). β-tubulin and calmodulin sequences confirmed its identification as *A. viridinutans*. Sequence divergence within our isolates is consistent with findings from previous studies demonstrating intraspecific genetic variability within the *A. viridinutans* species (*10*–*12*).

*A. viridinutans* was originally isolated from rabbit dung in Australia and has since been identified in soil samples globally (*12*,*13*). Although *A. viridinutans* has been reported from retrospective analysis of culture collections (*9*–*11*), its clinical relevance has not been defined. These cases demonstrate that *A. viridinutans* can cause a distinct form of aspergillosis, characterized by chronicity, propensity to spread in a contiguous manner across anatomical planes, and relative refractoriness to antimycotic drugs. In contrast, invasive aspergillosis affecting neutropenic chemotherapy or transplant patients is typically an acute disease with predilection for angioinvasion and hematogenous dissemination; successful response to antifungal therapy most commonly occurs within the first 6 weeks of treatment (*14*). However, *A. viridinutans* infections in 2 patients with distinct primary immunodeficiencies suggest that these clinical phenomena reflect inherent pathogenic features of the mold, rather than manifestations due to a specific immune defect. A similar constellation has been described for another cladistically related *fumigati*-mimetic, *Neosartorya udagawae*, in patients with chronic granulomatous disease or myelodysplasia (*15*), which may suggest pathogenetic differences between subgroups within section *Fumigati*. Thus, genotypic-based identification of *fumigati*-mimetic fungi may have implications for clinical course and management.
